# Postoperative empyema following lung cancer surgery

**DOI:** 10.18632/oncotarget.25629

**Published:** 2018-07-03

**Authors:** Noriyuki Matsutani, Katsuo Yoshiya, Masayuki Chida, Hirozo Sakaguchi, Takuma Kikkawa, Hiroki Fukuda, Nobumasa Takahashi, Noriyoshi Sawabata, Hirotoshi Horio, Nobuhiko Seki, Masafumi Kawamura

**Affiliations:** ^1^ Department of Surgery, Teikyo University School of Medicine, Tokyo, Japan; ^2^ Department of Thoracic Surgery, Niigata Cancer Center Hospital, Niigata, Japan; ^3^ Department of General Thoracic Surgery, Dokkyo Medical University, Tochigi, Japan; ^4^ Department of General Thoracic Surgery, Saitama International Medical Center, Saitama Medical University, Saitama, Japan; ^5^ Department of Surgery I, Tokyo Women's Medical University, Tokyo, Japan; ^6^ Department of General Thoracic Surgery, Saitama Medical Center, Saitama Medical University, Saitama, Japan; ^7^ Department of Thoracic Surgery, Saitama Cardiovascular and Respiratory Center, Saitama, Japan; ^8^ Department of Thoracic and Cardiovascular Surgery, Nara Medical University, Nara, Japan; ^9^ Department of General Thoracic Surgery, Tokyo Metropolitan Cancer and Infectious Diseases Center Komagome Hospital, Tokyo, Japan

**Keywords:** empyema, lung cancer, postoperative infection, surgery, mortality

## Abstract

Postoperative empyema following lung cancer surgery is a serious complication. Occurrence rate of postoperative empyema following lung cancer surgery, patient background, surgical procedures, date of empyema onset, treatment, and prognosis of 4772 patients who underwent lung cancer surgery between 2008 and 2012 were investigated.

Postoperative empyema following lung cancer surgery was found in 43 patients (0.9%). The occurrence rate of postoperative empyema was significantly higher in patients with the following factors: male gender, extended surgery such as pneumonectomy, bi-lobectomy and thoracotomy, squamous cell carcinoma, and an advanced pathologic stage of II and above. Chest drainage, video-assisted thoracic surgery debridement, fenestration, and thoracoplasy were performed, where 29 patients were cured (67.5%) and 5 patients (11.6%) died from thoracic empyema-related complications. Nine patients were not cured and died due to cancer or other diseases during treatment. When comparing cured and non-cured patients, it is indicated that squamous cell carcinoma, administration of steroids, history of interstitial pneumonia, presence of bronchial stump fistula, exacerbation of interstitial pneumonia and presence of non-fermenting Gram-negative bacilli led to a significantly low prognosis. The five-year overall survival rate was 34.9%.

## INTRODUCTION

Empyema is the main cause of death and causes serious complications within 30 days following lung cancer surgery [1]. Moreover, postoperative empyema prolongs hospitalization and postpones subsequent treatment. It has been reported that the occurrence rate for development of postoperative empyema before 2000 was between 3.7 and 13.6% [2–8]. In recent years, due to progress in perioperative management as well as the use of perioperative prophylactic antibiotics, the occurrence rate of empyema is approximately 1% [9–11]. On the other hand, the risk in developing postoperative complications is increasing due to increased comorbidity caused by perioperative chemotherapy and advanced patient age [6]. Reports on factors inducing postoperative empyema and treatments and prognosis for postoperative empyema have been scarcely available. In this retrospective multicenter study, the risk factors for developing postoperative empyema following lung cancer surgery and its prognosis were conducted.

## RESULTS

Of 4772 patients, 3035 patients were male, and 1737 patients were female. There were 3471 patients under the age of 75 years and 1301 patients over the age of 75 years. The number of patients who underwent lobectomy was the largest, being 3326, followed by segmentectomy, wedge resection, bi-lobectomy, and pneumonectomy, where the numbers were 651, 585, 111, and 91, respectively. Thoracotomy and video-assisted thoracic surgery (VATS) were performed on 3405 and 1367 patients, respectively. Sleeve resection and induction therapy were performed on 118 and 114 patients, respectively. Three thousand two hundred ninety patients were in Stage I and 652 patients in Stage II, while there were 827 patients in Stages III and IV. Histological analysis indicated that 3322 patients had adenocarcinoma and 1046 patients had squamous cell carcinoma (Table 1).

**Table 1:** Patient characteristics

Variable	Lung cancer surgery (*n* = 4772)	Empyema (*n* = 43)	%	*p*-value
Total number	4772	43	0.90	
Sex				
Male	3035	37	1.22	0.002
Female	1737	6	0.35	
Age				
<75	3471	29	0.84	0.39
≥75	1301	14	1.08	
Procedure				
Pneumonectomy	91	8	8.79	<0.0001
Bi-lobectomy	111	3	2.70	
Lobectomy	3326	26	0.78	
Segmentectomy	651	5	0.77	
Wedge resection	585	1	0.17	
Method				
Thoracotomy	3405	39	1.15	0.005
Video-assisted thoracic surgery	1367	4	0.29	
Sleeve resection	118	2	1.69	0.355
Induction therapy	114	2	1.75	0.329
Pathologic stage				
I	3290	21	0.64	0.005
II	652	11	1.69	
III, IV	827	11	1.33	
Histology				
Squamous cell ca.	1046	16	1.53	0.009
Non-squamous cell ca.	3726	27	0.72	
adenocarcinoma	3322	21	0.63	
large cell ca.	110	1	0.91	
small cell ca.	76	1	1.32	
adenosquamous ca.	67	1	1.49	
pleomorphic ca.	62	2	3.23	
others	97	1	1.03	

Postoperative empyema was found in 43 patients (43/4772, 0.90%), where 37 patients were male and 6 patients were female, showing a significant gender difference (*p* = 0.002), although no difference was found by age. Surgical procedure which caused the highest postoperative empyema occurrence rate was pneumonectomy followed by bi-lobectomy, which were significantly higher than that of lobectomy, segmentectomy, and wedge resection (*p* < 0.001). Moreover, the number of patients who underwent thoracotomy showed a higher occurrence rate of postoperative empyema compared with those who underwent VATS (*p* = 0.005). There was no significant difference in the occurrence rate of postoperative empyema between patients who underwent sleeve resection, or patients who underwent induction therapy. Histological analysis indicated that a higher frequency of postoperative empyema was found in patients with squamous cell carcinomacompared with those with non-squamous cell carcinoma (*p* = 0.009). Patients in Stage I developed fewer postoperative empyema compared with those in Stages II, III, or IV (*p* = 0.005) (Table 1).

Among the 43 patients with postoperative empyema, the causes of empyema were bronchial stump fistula in 14 patients, retrograde infection due to a chest tube in 12 patients, bronchopleural fistula in 11 patients, exacerbation of interstitial pneumonia in 4 patients, intraoperative contamination in 3 patients, surgical site infection in 3 patients, and pneumonia in 3 patients. The date when postoperative empyema was first observed was between the 4th and 425th day (average being 43.9th day) while 29 patients developed postoperative empyema early within 30 days and 14 patients developed late after 30 days. Pleural effusion samples were collected from 43 patients to examine the presence of bacteria by culturing. Of 43 samples, 37 samples contained a total of 46 types of bacteria, where MRSA was found in 10 samples, MSSA was in 9 samples, *Pseudomonas* was in 6 samples, *Enterococcus* was in 4 samples, and other types of bacteria were found in 16 samples (Table 2).

**Table 2:** Prognostic factors: univariate analysis

Variable	*n*	Cure (*n* = 29)	Non-cure (*n* = 14)	*p*-value
Sex				
Male	37	24	13	0.371
Female	6	5	1	
Age				
<75	29	20	9	0.759
≥75	14	9	5	
Method				
Thoracotomy	39	26	13	0.735
Video-assisted thoracic surgery	4	3	1	
Pathologic stage				
I	21	13	8	0.449
II, III, IV	22	16	6	
Histology				
Squamous cell ca.	16	7	9	0.011
Non-squamous cell ca.	27	22	5	
Procedure				
Pneumonectomy, Bi-lobectomy	11	5	6	0.071
Others	32	24	8	
Pre-operative complications				
Hypertension	14	8	6	0.371
Chronic obstructive pulmonary disease	6	4	2	0.965
Administration of steroid	5	1	4	0.016
Other organs cancer	4	3	1	0.735
Diabetes mellitus	4	2	2	0.434
Ischemic heart disease	3	1	2	0.191
Cerebrovascular disease	3	2	1	0.976
Interstitial pneumonia	2	0	2	0.037
Date of onset				
<30 days	29	20	9	0.759
≥30 days	14	9	5	
Cause of empyema				
Bronchial stump fistula	14	6	8	0.017
Retrograde infection by chest tube	12	12	0	0.005
Bronchopleural fistula	11	7	4	0.755
Exacerbation of interstitial pneumonia	4	0	4	0.003
Intraoperative contamination	3	2	1	0.976
Surgical site infection	3	3	0	0.212
Pneumonia	3	3	0	0.212
Microbiology				
MRSA	10	5	5	0.179
MSSA	9	8	1	0.123
Pseudomonas aeruginosa	6	4	2	0.965
Enterobacteriaceae	4	1	3	0.057
Enterococci	3	2	1	0.976
Aspergillus	2	1	1	0.590
Nontuberculous mycobacteria	1	1	0	0.482
Haemophilus influenzae	1	1	0	0.482
Anaerobe	4	4	0	0.145
Gram-positive coccus	3	3	0	0.212
Non-fermenting Gram-negative bacilli	2	0	2	0.037
Corynebacterium spp	1	0	1	0.145
Culture negative	6	3	3	0.326

Abbreviations: MRSA: Methicillin-resistant Staphylococcus aureus; MSSA: Methicillin-susceptible Staphylococcus aureus.

**Figure 1:**
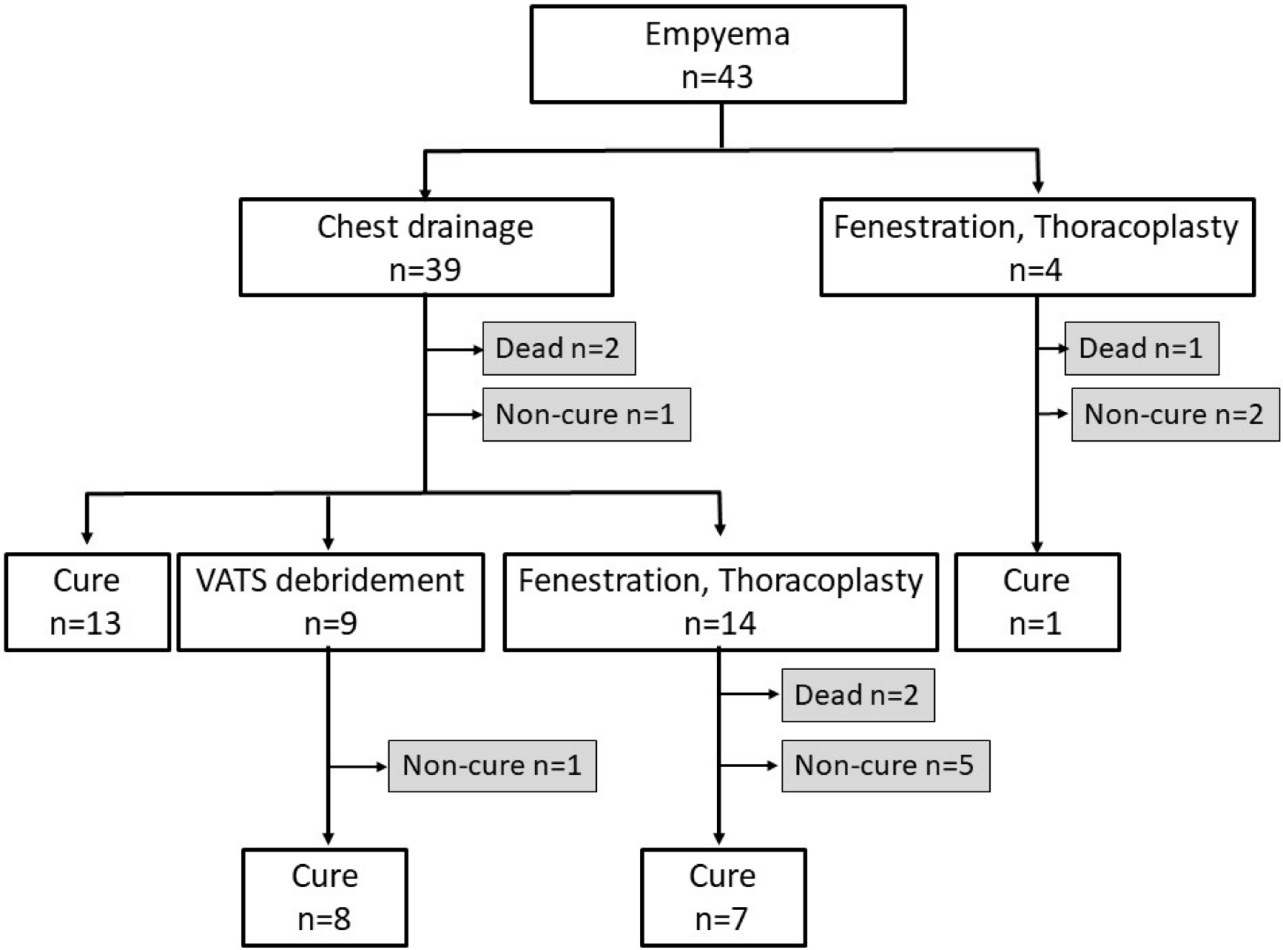
Treatment flowchart of postoperative empyema VATS: video-assisted thoracic surgery.

Of 43 postoperative empyema patients, 39 patients underwent chest drainage as an initial treatment, and while 13 of these were cured, 2 died due to empyema-related diseases and 1 died due to other disease during treatment. VATS debridement was added to the initial chest drainage for 9 patients and fenestration and thoracoplasy were added to 14 patients. Four patients underwent fenestration and thoracoplasy as the initial treatment, and while 1 of them was cured, 1 died from empyema-related causes and 2 were not cured. Of 9 patients who received VATS debridement as a secondary treatment, 8 patients were cured while 1 patient was not cured. Moreover, of 14 patients who received fenestration and thoracoplasy as a secondary treatment, 7 patients were cured while 2 patients died from empyema-related causes and 5 patients were not cured. Thus, 5 patients with postoperative empyema died from empyema-related causes resulting in a mortality rate of 11.6% (Figure 1).

Twenty-nine patients (67.5%) were cured while of the 14 non-cured patients, there were 5 patients who died from empyema-related causes. Six patients died from cancer during empyema treatment, 2 patients died from other diseases, and 1 patient committed suicide.

When comparing cured and non-cured patients, it is indicated that gender, age, type of surgery, and pathologic stage did not impact prognosis of the disease; however, squamous cell carcinoma, administration of steroid, history of interstitial pneumonia, presence of bronchial stump fistula, exacerbation of interstitial pneumonia and presence of non-fermenting Gram-negative bacilli led to a significantly low prognosis (Table 2).

Risk factors selected by univariate analysis were subjected to multivariate analysis; however, no independent risk factors were found (Table 3).

**Table 3:** Prognostic factors: multivariate analysis of Cox proportional hazards model

Variable	risk ratio	95% CI	*p*-value
Cause of empyema			
Exacerbation of interstitial pneumonia	0	0.00	0.999
Bronchial stump fistula	0.371	0.064–2.165	0.558
Pre-operative complication			
Administration of steroid	0.188	0.008–4.311	0.296
Interstitial pneumonia	1.844	0.00	1
Histology			
Squamous cell ca.	0.325	0.053–1.982	0.223
Microbiology			
Non-fermenting Gram(-) bacilli	0	0.00	0.999

**Figure 2:**
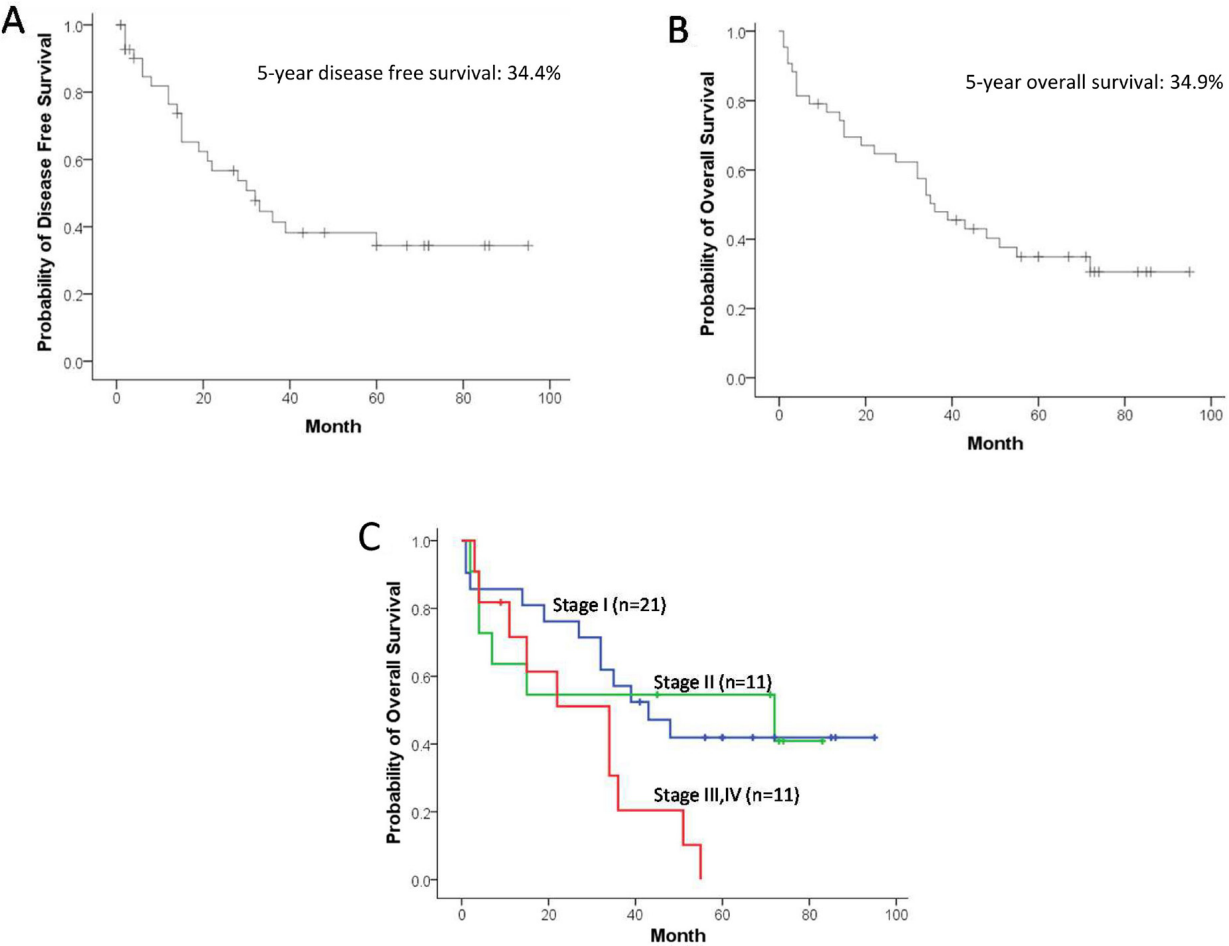
Cancer free survival curve (**A**), overall survival curve (**B**) and pathologic stage-specific survival curves, stage I (*n* = 21, blue), stage II (*n* = 11, green), and stage III and IV (*n* = 11, red) (stage I vs. II; *p* = 1.000, stage I vs. III and IV; *p* = 0.032, stage II vs. III and IV; *p* = 0.081) (**C**).

The 3-year and 5-year cancer free survival rates were 41.4% and 34.4%, respectively (Figure 2A). Regarding survival rates, the 3-year and 5-year overall survival rates were 47.9% and 34.9%, respectively, with an average observation period and observation median being 46.8 months and 36 months, respectively (Figure 2B). The 3-year overall survival rates by pathologic stage were 57.1% for stage I, 54.5% for stage II, and 20.5% for stage III and IV, while the 5-year overall survival rates were 41.9% for stage I, 54.5% for stage II, and 0% for stage III and IV. The difference in overall survival was statistically significant between stage I and stage III and IV (*p* = 0.032), whereas no significant differences were shown between stage I and stage II (*p* = 1.000), and between stage II and stage III and IV (*p* = 0.081) (Figure 2C).

## DISCUSSION

Reports regarding postoperative empyema following lung cancer surgery had been derived from a single institution where the lung cancer surgery numbers were between 199 and 1855 (Table 4) [2–8, 10, 11]. In this manuscript, 4773 patients who underwent lung cancer surgery at multiple centers were studied. Although Nagasaki reported that the occurrence rate of postoperative empyema following lung cancer surgery was 0.9% before 2000, it was commonly understood that the actual rate was 3.7–13.6%, while the rate after 2000 was reported to be approximately 1% (Table 4) [2–8, 10, 11]. The reasons for the improvement in the occurrence rate of empyema were thought to be derived from improvements in perioperative management [10].

**Table 4:** Literature on postoperative empyema in lung cancer surgery

Author (year)	Period	Institution	Surgery	Empyema	Incidence	Mortarity	5-year survival
Ruckdeschel (1972)	1952–1966	single	489	18	3.7%	n/a	50.0%
Brohee (1977)	1961–1972	single	349	45	12.9%	n/a	20.0%
Pastorino (1982)	1974–1977	single	199	27	13.6%	14.8%	28.0%
Nagasaki (1982)	1973–1980	single	961	9	0.9%	22.2%	n/a
Deslauriers (1994)	1988–1989	multiple	783	39	5.0%	n/a	n/a
Giorgio (1996)	1960–1984	single	460	38	8.3%	n/a	36.8%
Duque (1997)	1993–1994	multiple	605	27	4.5%	n/a	n/a
Shiono (2007)	1992–2003	single	1855	18	1.0%	n/a	n/a
Yamauchi (2013)	2002–2011	single	1673	27	1.6%	n/a	n/a
This study	2008–2012	multiple	4772	43	0.9%	11.6%	34.9%

Pneumonectomy can be a risk factor which can cause postoperative empyema [3, 5, 8]. Excluding the report published by Nagasaki where the rate of pneumonectomy was 8% [4], the rate of pneumonectomy as a surgical technique used before 2000 was extremely high, being 17.2–45.3% [5–8]. In recent years, the incidence of pneumonectomy has decreased to 1.9–4.7%; thus, the occurrence rate of postoperative empyema cases is decreasing as pneumonectomy is performed less frequently [10, 11]. Other risk factors such as gender being male [6, 10], being elderly [6, 10], induction therapy [6], and diabetes mellitus [8], are reported as risk factors which can cause postoperative empyema. It has been reported that decreased immunological response of patients due to advanced disease stage and extended surgery can impact the development of postoperative empyema [5].

In this study, the occurrence rate of postoperative empyema was significantly higher in patients with factors including male gender, extended surgical procedures such as pneumonectomy and bi-lobectomy, thoracotomy, squamous cell carcinoma, and an advanced pathologic stage of II and above. Patients at an advanced pathologic stage with squamous cell carcinoma require pneumonectomy; thus, it is thought that these conditions may result in the development of postoperative empyema following lung cancer surgery. In recent years, it is considered that the increased use of minimally invasive surgery such as VATS, instead of thoracotomy, may help to reduce the number of postoperative empyema occurrences [12, 13].

Reports on factors inducing postoperative empyema and treatments and prognosis for postoperative empyema have been scarcely available; thus, this study is the first to report on these questions. According to this study, it was discovered that empyema in 58.2% of patients was due to fistulas such as bronchial stump fistula and bronchopleural fistula. Among these fistulas, the prognosis for bronchial stump fistula is significantly poor and the fistula is difficult to cure. The next highest factor, 27.9% of empyema patients, which induces empyema is retrograde infection due to a chest tube; however, all of them were cured. On the other hand, none of the empyema patients with interstitial pneumonia were cured. Thirty-nine (90.7%) of the empyema patients underwent chest drainage as an initial treatment of empyema while 4 patients (9.3%) underwent fenestration from the beginning. While thirteen empyema patients (30.2%) were cured by chest drainage alone, another 23 chest drainage patients required additional treatment by VATS debridement or fenestration.

Empyema is a fatal condition in itself where the death rate due to empyema is 10–16% [14–16]. The death rate of patients with postoperative empyema following lung cancer surgery was 14.8–22.2% according to reports published in 1982 [4, 5]. In this study, the death rate of patients with postoperative empyema was 11.6%, the rate being approximately the same as empyema patients who did not undergo surgery.

The death rate for patients following lung cancer surgery in Japan was 1.6% before 2002, where 33% of such deaths were caused by bronchial stump fistula and empyema (19/3270; 0.581%) [1]. The death rate of patients following lung cancer surgery is 0.7–0.8% since 2010, where 6.2–9.1% of such deaths are caused by bronchial stump fistula and empyema (41/32,801; 0.125% [12], 20/38,085; 0.053% [13], this study 5 of 4772; 0.105%). Thus, the death rate following lung cancer surgery has decreased.

The risk factors which impact prognosis of empyema, including a cancer type being squamous cell carcinoma, administration of steroids, history of interstitial pneumonia, presence of bronchial stump fistula, exacerbation of interstitial pneumonia, and presence of non-fermenting Gram-negative bacilli possibly turning to drug resistance, were identified by univariate analysis. No independent risk factors were identified by multivariate analysis. However, the reasons may be due to the small patient number and each factor playing a confounding factor.

Postoperative empyema was thought to increase immune response, which improves the prognosis of lung cancer [2, 17–20]. This hypothesis came from an initial discovery by Dr. Graham where he performed the first successful pneumonectomy on a patient with lung cancer who survived longer than 30 years following postoperative empyema [21]. On the other hand, there are reports that empyema does not improve the prognosis of patients with lung cancer [3, 5]. Neither case is convincing since patient numbers were small and controls were not adequate. In this study, the 5-year overall survival rate for all postoperative empyema was 34.9%, where by disease stage was 41.9% at stage I, 54.5% at stage II, and 0% at stages III and IV. In a prognosis study conducted in Japan, the 5-year survival rate of 11,663 patients by disease stage following lung cancer surgery was 86.8% at stage IA, 73.9% at stage IB, 61.6% at stage IIA, 49.8% at stage IIB, 40.9% at stage IIIA, 27.8% at stage IIIB, and 27.9% at stage IV [22]. The number of patients with postoperative empyema was small and therefore it is not adequate to compare data from the 2 different studies; however, it may not be true that postoperative empyema improves prognosis of patients who underwent lung cancer surgery.

Postoperative empyema following lung cancer surgery is a serious complication. However, no reports on treatments of empyema and prognosis of patients with empyema have been published. This study is retrospective. However, the data were collected from 4772 patients who underwent lung cancer surgery and the actual cause, treatment, and prognosis of empyema were reported.

There were some limitations of this study. Detailed analysis was possible for only 43 patients with postoperative empyema, however, detailed information regarding past history, complications, operative time, blood loss during surgery, coverage of the bronchial stump and prognosis of other patients who underwent lung cancer surgery was difficult to obtain. Because of the multi-institutional study, the surgical procedures, and the perioperative management were different among institutions. Although the occurrence rate, inducing factors and prognosis for postoperative empyema were indicated, to assess and plan for patients with the risk preoperatively cannot be devised from this study. Based on this study, future prospective studies of postoperative empyema are anticipated.

Lung cancer surgery is becoming minimally invasive, reducing the occurrence rate of postoperative empyema following lung cancer surgery to 0.9%. This report determined that male gender, extended surgery, open thoracotomy, squamous cell carcinoma, and advanced stage were risk factors inducing postoperative empyema following lung cancer surgery. The cure rate was 67.5%, and the mortality rate was 11.6%.

## MATERIALS AND METHODS

This study was approved by the Institutional Ethics Review Board (Teikyo University Review Board 12–152) and other institutions, and the need for informed consent from patients was waived provided that patient data remained anonymous.

### Patients

This study was conducted on 4772 patients who underwent thoracic surgery to treat primary lung cancer between January, 2008 and December, 2012 at 9 institutions in Japan. The surgical indications and procedures for lung cancer were determined by each institution. All the participating institutions were certified by The Japanese Association for Chest Surgery, and respiratory surgery specialists performed treatment according to Lung Cancer Clinical Practice Guidelines by The Japan Lung Cancer Society, where patients with performance status 0 and 1 were subjects undergoing surgery. Perioperative management at each institution was approximately the same. An antibiotic, Cefazolin at 7 institutions and Piperacillin at 2 institutions, was administered prophylactically to patients beginning immediately before surgery. Administration of antibiotics was ended on the day after surgery at 2 institutions, on the next day after surgery at 3 institutions, on the 2nd day after surgery at 2 institutions, and on the 3rd day after surgery at 2 institutions. Antibiotics and duration of antibiotics used were modified depending on the symptoms of infection. A chest tube (size: 19 –28Fr) was installed in the pleural space at the time of chest closure. The chest tube was removed when air leakage, hemothorax, and chylothorax were not observed and when the amount of pleural effusion was less than 300 mL per day. No institution conducted bacterial culture from the chest tube tip.

### Definitions

Purulent effusion collection in the postoperative thoracic cavity with over Grade IIIa of Clavien-Dindo classification is defined as empyema. The bacterial culture results of purulent effusion were not accounted for. Cancers were pathologically classified according to the eighth edition of the TNM classification for lung cancer according to the international association for the study of lung cancer [23]. Video-assisted thoracic surgery was defined as surgery with a thoracoscopic field of view, and a chest metallic retractor was not used, in this study.

### Data collection

The following data were collected from patients with postoperative empyema after lung cancer surgery: gender, age, pre-operative complications, treatment for the lung cancer, date of surgery, type of surgery, type of histology, pathologic stage (I, or II/III/IV), date of empyema confirmed, cause of empyema, bacterial test of pleural effusion, treatment for the empyema, date of lung cancer recurrence, and disease status during follow-up. Patients were selected by each institution from those whose causes of empyema were intraoperative contamination, infection from surgical site, retrograde infection due to a chest tube, bronchial stump fistula, bronchopleural fistula, pneumonia, interstitial pneumonia, unknown, and others. Intraoperative contamination is the contamination by exposure of pulmonary contents and sputum during operation, and retrograde infection due to a chest tube is the contamination of chest drain penetration site or back flow of contents of drainage bag. The doctor in charge raised the causes which seemed appropriate. Data regarding gender, age, type of surgery, type of histology, and pathologic stage were collected from the 4772 patients included in the study.

### Statistical analysis

Chi-square and Student’s *t*-test were used to compare the percentages and mean values. Overall survival and disease-free survival were analyzed using the Kaplan–Meier method. Univariate and multivariate Cox proportional hazards regression analyses were used to evaluate potential prognostic factors. Data were analyzed using version 24 of the IBM SPSS software (IBM Corporation, Somers, NY, USA). A *p*-value < 0.05 was considered statistically significant.

## Abbreviations

VATS: video-assisted thoracic surgery
